# Targeting Cellular Metabolism Chemosensitizes the Doxorubicin-Resistant Human Breast Adenocarcinoma Cells

**DOI:** 10.1155/2015/453986

**Published:** 2015-10-19

**Authors:** Shulan Ma, Rongfei Jia, Dongju Li, Bo Shen

**Affiliations:** ^1^Training Center of Medical Experiments, Basic Medical School, Fudan University, Shanghai 200032, China; ^2^Department of Oncology, Shanghai Xuhui Central Hospital, Shanghai 200031, China; ^3^Institute of Radiation Medicine, Fudan University, Shanghai 200032, China

## Abstract

Metabolic energy preferentially produced by glycolysis was an advantageous metabolic phenotype of cancer cells. It is also an essential contributor to the progression of multidrug resistance in cancer cells. By developing human breast cancer MCF-7 cells resistant to doxorubicin (DOX) (MCF-7/MDR cells), the effects and mechanisms of 2-deoxy-D-glucose (2DG), a glucose analogue, on reversing multidrug resistance were investigated. 2DG significantly inhibited the viability of MCF-7/MDR cells and enhanced DOX-induced apoptosis by upregulating protein expression of AMPK*α*, P53, and caspase-3. The study demonstrated that energy restriction induced by 2DG was relevant to the synergistic effect of 2DG and DOX. The proteins of multidrug gene (the MDR-related protein, MRP1) and P-glycoprotein (P-gp) in MCF-7/MDR cells were downregulated after exposure to 2DG, accompanied with the suppression of the activity of ATP-dependent drug-efflux pump and transmembrane transporter, increasing the intracellular accumulation of DOX to reverse the chemoresistance in multidrug cancer cells.

## 1. Introduction

Scientific literature indicates that cancer cells predominantly generate energy by a high rate of glycolysis rather than the Krebs cycle of mitochondrial metabolism even when oxygen is abundant, which is called Warburg's effect [1]. Glycolytic activity is an inefficient way for energy production. Elevated amount of glucose is consumed to meet the rapid progression of the tumor. Based on this, the abnormal uptake of 2-[^18^F]fluoro-2-deoxy-D-glucose (FDG) has been utilized in imaging by positron emission tomography/computed tomography (PET/CT) [2]. Furthermore, due to the accumulation of lactic acid from Warburg's effect, the acidification of extracellular microenvironment favors the progression and metastases of tumor via upregulation of metallo-proteinase and cysteine proteinase activity and secretion [3]. It has been suggested that the metabolic characteristic of cancer cells facilitates cancer cells' survival and contributes to their resistance to conventional cancer therapies, such as chemotherapy and radiotherapy [4].

Breast cancer is one of the common malignant tumors affecting adult women. Doxorubicin (DOX) has been considered as one of the potent first-line chemotherapeutic agents for breast cancer treatment due to its high efficacy and tolerance. However, the development of multidrug resistance (MDR) is an unfavorable factor in the poor prognosis for breast cancer. Meantime, the development of MDR is accompanied with the overexpression of ATP-dependent drug-efflux pump, such as p-glycoprotein (P-gp), multidrug resistance protein 1 (MRP1), and the breast cancer resistance protein (BCRP), which is thought to be an essential obstacle to reducing intracellular drug accumulation in tumor eradication [5].

In the current study, we developed DOX-resistant MCF-7 cells and examined the antiproliferative effect of DOX when the cellular metabolism was modulated with a glucose inhibitor. The results showed that energy restriction sensitized the DOX-resistant MCF-7 cells and enhanced the apoptosis induced by DOX. Since the glucose inhibitor induced a dramatic reduction in cellular ATP levels, the possible mechanism may be related to the functions of drug-efflux pumps, where the activity of transmembrane transporters regulates the energy restriction.

## 2. Materials and Methods

### 2.1. Drugs

Doxorubicin (DOX) and 2-deoxy-D-glucose (2DG) were purchased from Sigma-Aldrich Co. (St. Louis, MO, USA). DOX was dissolved in water as a 1 M stock solution. 2DG was dissolved in phosphate-buffered saline (PBS) to prepare a stock solution of 1 M. Drugs were serially diluted with culture medium before use.

### 2.2. Cell Line

Human breast carcinoma cell line MCF-7, obtained from Shanghai Institutes for Biological Science, Chinese Academy of Sciences (Shanghai, China), was maintained in Dulbecco's Modified Eagle Medium (HyClone, Beijing, China) containing glucose (4.5 g/L) and supplemented with penicillin (100 units/mL), streptomycin (100 *μ*g/mL), glutamate (2 mM), and 10% fetal bovine serum (Gibco Invitrogen, Grand Island, NY, USA). MCF-7/MDR cells, resistant to DOX, were derived by stepwise selection with DOX and were maintained in the presence of 1 *μ*M DOX. Cells were grown in normal culture medium for 1 week before each experiment. Cells were maintained in a humidified atmosphere of 5% CO_2_ in air at 37°C.

### 2.3. Cell Viability Assay

The quantity of viable cells after treatment with various agents was determined with the Cell Counting Kit-8 (CCK8) (Dojindo, Shanghai, China) according to the manufacturer's protocol. Briefly, cells were plated into 96-well plates (100 *μ*L, 5 × 10^3^/well) and allowed to adhere overnight and were treated with various concentrations of drugs for 24 h. The culture medium was replaced with fresh medium containing CCK8 solution (10% V/V) and the plates were incubated for additional 4 h at 37°C. The absorbance at 450 nm was measured on a microplate reader (Bio-Tek, USA). The cell viability was calculated by the formula

(1) $$\begin{array}{c} cell\;\;viability\left(\;\%\;\right)=\frac{\left({OD}_{treatment}-{OD}_{blank}\right)}{\left({OD}_{control}-{OD}_{blank}\right)}\times 100\%. \end{array}$$

### 2.4. Apoptosis Analysis

Culture medium containing floating cells was removed and retained. The monolayer cells were rinsed with PBS and harvested with trypsin (without EDTA). The cells were then pooled with the floating cells, washed, and resuspended in 100 *μ*L binding buffer supplemented with Annexin V-FITC (5 *μ*L) and PI (5 *μ*L). All samples were incubated in the dark room. The stain was stopped by adding 300 *μ*L binding buffer. Apoptotic cells were analyzed by a flow cytometer (Gallios, Beckman Coulter, USA). At least 10,000 cells from each sample were analyzed.

### 2.5. Determination of Intercellular ATP

Intracellular ATP was determined as previously described using a Bioluminescence Detection Kit for ATP (Promega Co., USA) [6]. Briefly, cells were treated with 2DG (from 5 mM to 80 mM) for 24 h. Whole-cell extracts of 1 × 10^5^ cells were prepared in lysis buffer. Pellet debris was centrifuged briefly and 10 *μ*L of supernatant was mixed with 100 *μ*L Luciferase Assay Reagent of ATP assay to measure the light produced. Fluorescence light emission was measured with a microplate reader (Bio-Tek, USA).

### 2.6. Estimation of Na^+^, K^+^-ATPase Activity

According to the method described previously [7], determination of Na^+^-K^+^-ATPase activity was carried out with some modifications. Aliquots of cell extract were prepared by ultrasonication. Samples were added to an enzyme reaction mixture (100 mM Tris-HCl buffer, 125 mM NaCl, 75 mM KCl, 7.5 mM MgCl_2_, and 10 mM Na_2_ATP, at pH 7.5). The reaction was incubated at 28°C for 20 min followed by immediate ice bath to stop the reaction. Released inorganic phosphate (Pi) was measured at 650 nm in the color reagent (1% Tween 20 and 1% ammonium molybdate in 0.9 M H_2_SO_4_).

### 2.7. Rhodamine 123 (Rh 123) Accumulation Study

The cellular accumulation of fluorescent dye Rh 123 was used to examine the effects of 2DG on the functional activity of P-gp according to the method of Fontaine et al. [8]. MCF-7/MDR cells at a density of 1 × 10^5^ cells/mL in exponential growth were preincubated with various concentrations of 2DG or combined with 30 *μ*M DOX for 4 h. And then, the cells were incubated in the presence of 10 *μ*M Rh123 at 37°C for up to 120 min. After removing the dye, the fluorescence intensity of 10,000 cells was measured using flow cytometry.

### 2.8. Real-Time Polymerase Chain Reaction Analysis

Quantitative real-time reverse transcription polymerase chain reaction (qRT-PCR) was employed for gene expression analyses. Amplification of genes was performed using SYBR Green Real Master Mix (Tianjin, China). Sense and antisense primers used for amplification in this study were as follows: 5′-ATCGCCGTGTTTGGCTACTCC-3′ and 5′-AAGCGGTTCACCAGGTTCCC-3′ for MRP1; 5′-GGGTGGTGTCACAGGAAGAGATT-3′ and 5′-GGCTGTCTAACAAGGGCACGA-3′ for P-gp; and 5′-GGAGTCCACTGGCGTCTTC-3′ and 5′-GCTGATGATCTTGAGGCTGTTG-3′ for GAPDH. All real-time experiments were run in triplicate and a mean value was used for the determination of mRNA levels. Relative mRNA expression levels for MRP1, P-gp, and GAPDH were determined using the 2^−ΔΔ*Ct*^ method and normalized to the GAPDH.

### 2.9. Western Blot Analysis

After treatment, the cell lysate was prepared with RIPA lysis buffer. The protein content of the extracts was determined using BCA protein assay kit (Beyotime, China). An equal amount of total protein was subjected to 10% SDS-PAGE and transferred to PVDF membrane (Millipore, Bedford, USA). The following antibodies were used to probe the corresponding proteins: anti-phospho-AMPK*α* (CST, USA), anti-AMPK*α* (CST, USA), caspase-3 (Epitomics, USA), anti-p53 (Epitomics, USA), anti-phospho-p53 (Epitomics, USA), and anti-GAPDH (Beyotime, China). GAPDH was used for the loading control. The protein bands were visualized using the ChemiDoc XRS system (Bio-Rad Laboratories, USA). The protein level was determined with Quantity One software (Bio-Rad Laboratories).

### 2.10. Statistics

Experimental data were presented as mean with standard deviation for at least three independent experiments and analyzed with the SPSS 13.0 software. The difference between groups was assessed using Student's *t*-test and *P* < 0.05 was considered to be significant.

## 3. Results

### 3.1. 2DG Inhibited the Proliferation of DOX-Resistant Breast Cancer Cells by Depleting Intracellular ATP Supplement

**Figure 1:**
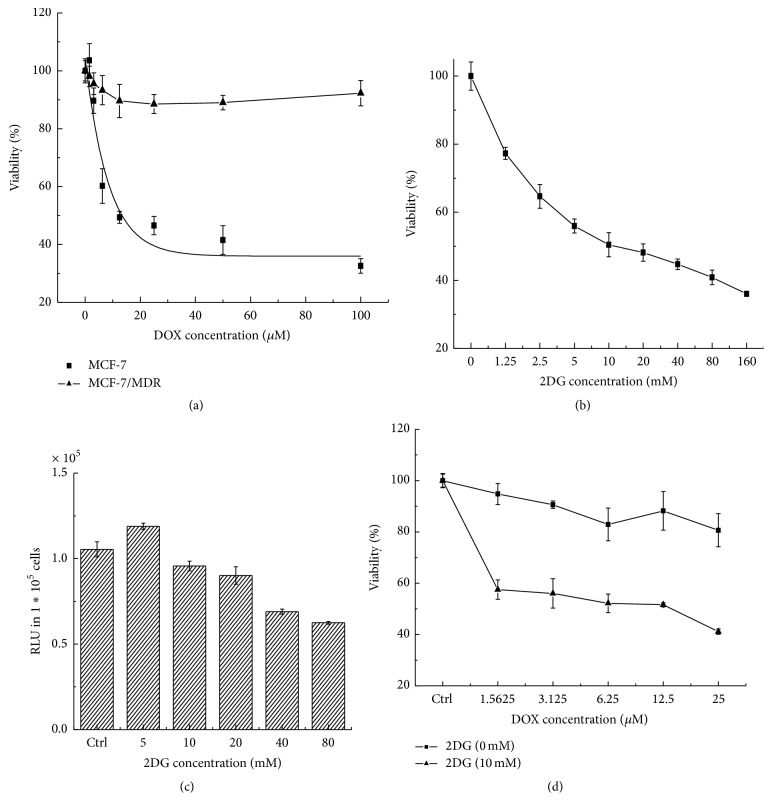
Energy restriction induced by 2DG inhibited the proliferation of MCF-7/MDR cells. (a) The establishment of MCF-7/MDR cells. (b) 2DG depressed the proliferation of MCF-7/MDR cells in a dose-dependent manner. (c) 2DG depleted the level of intercellular ATP. (d) 2DG enhanced the cytotoxicity of DOX.

The resistance of MCF-7/MDR cell line towards DOX was testified with CCK8 assay. The results revealed that IC_50_ values of DOX for MCF-7 and MCF-7/MDR cells were 23.52 *μ*M and 678.15 *μ*M, respectively (Figure 1(a)). It confirmed that MCF-7/MDR was 300-fold more resistant to the effects of DOX, compared to MCF-7 cells. Furthermore, MCF-7/MDR cells exhibited obvious growth depression towards 2DG in a concentration dependent manner (Figure 1(b)). To examine whether the effect is attributed to the lack of cellular ATP, intracellular ATP levels were detected when MCF-7/MDR cells were exposed to 2DG at various concentrations for 24 h. Except that 5 mM 2DG stimulated the generation of cellular ATP, a dose-dependent decrease in the ATP level was found from 10 mM to 80 mM (Figure 1(c)). To verify the interaction between DOX and 2DG, 10 mM 2DG was added to sensitize MCF-7/MDR cells to DOX. The results showed that DOX IC_50_ in the presence of 10 mM 2DG declined to 1.56 *μ*M, which led to a more than 400-fold increase of DOX cytotoxicity (Figure 1(d)). It was revealed that energy restriction sensitized MCF-7/MDR cells towards DOX.

### 3.2. Intracellular Energy Restriction Reversed the Resistance of Cells towards DOX by Depressing Drug-Efflux Transporters

**Figure 2:**
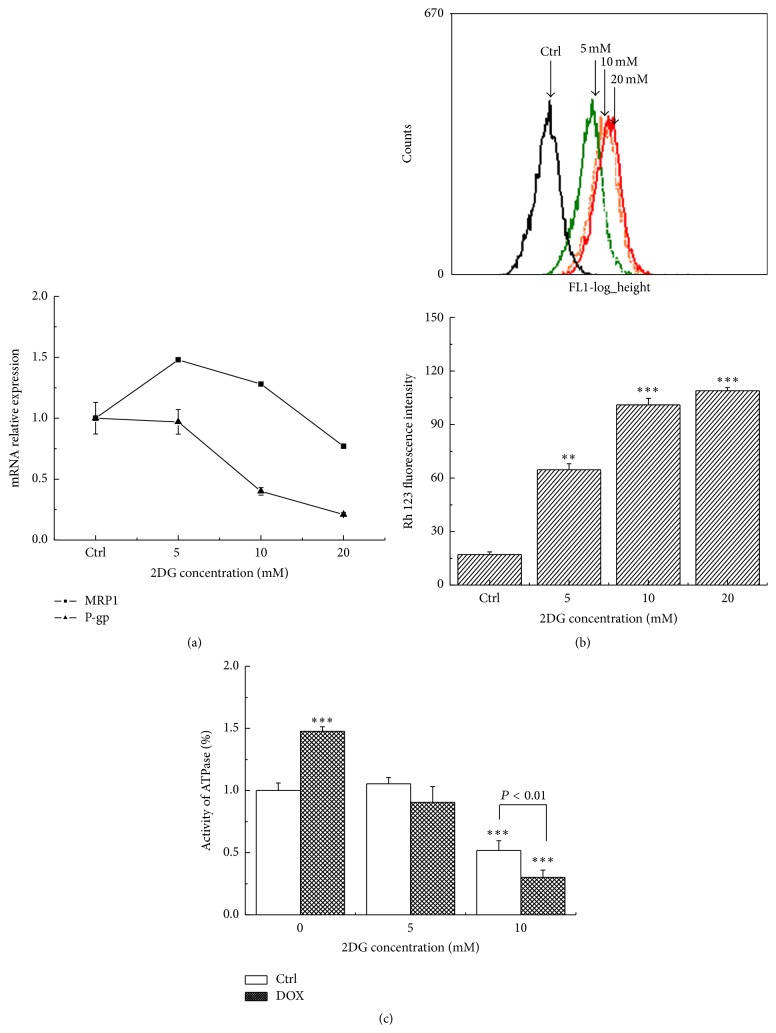
Energy restriction induced by 2DG depressed the activity of transmembrane transporter system. (a) 2DG decreased the mRNA expression of multidrug gene, MRP1, and P-gp. (b) The intensity of Rh 123 fluorescence was detected with flow cytometer. ^*∗∗*^ *P* < 0.01; ^*∗∗∗*^ *P* < 0.001 versus control group. (c) The activity of cellular Na^+^-K^+^-ATPase was examined. ^*∗∗∗*^ *P* < 0.001 versus control group.

The occurrence of multidrug resistance has been widely recognized as the overexpression of efflux transporters, such as P-glycoprotein (P-gp) and the MDR-related protein (MRP1). To determine whether changes in the mRNA levels of these genes correlate with the effect of 2DG in MCF-7/MDR cells, real-time PCR was carried out to evaluate the differences in expression level of the chosen genes. As shown in Figure 2(a), pretreatment MCF-7/MDR cells with 20 mM 2DG decreased MDR1 expression by 23 ± 0.1% and P-gp expression by 79 ± 1.5%, compared to the control group. In parallel, we measured the cellular accumulation of Rd 123 to evaluate the function of P-gp (Figure 2(b)). As expected, 2DG treatment significantly increased Rd 123 accumulation. The intensity of Rd 123 fluorescence was remarkably increased upon increasing the concentration of 2DG from 64.66 ± 3.37% at 5 mM to 108.94 ± 1.82% at 20 mM.

In this study, we also examined the activity of cellular Na^+^-K^+^-ATPase after the MCF-7/MDR cells were exposed to 2DG. As shown in Figure 2(c), the independent administration of 30 *μ*M DOX activated the Na^+^-K^+^-ATPase significantly. However, the addition of 2DG depressed its activity up to 50% at the higher treatment concentration examined (10 mM) compared with the control group, and the inhibition was up to 70% in the presence of 30 *μ*M DOX.

### 3.3. Energy Depletion Enhanced the Cytotoxicity of Doxorubicin by Inducing Cell Apoptosis

**Figure 3:**
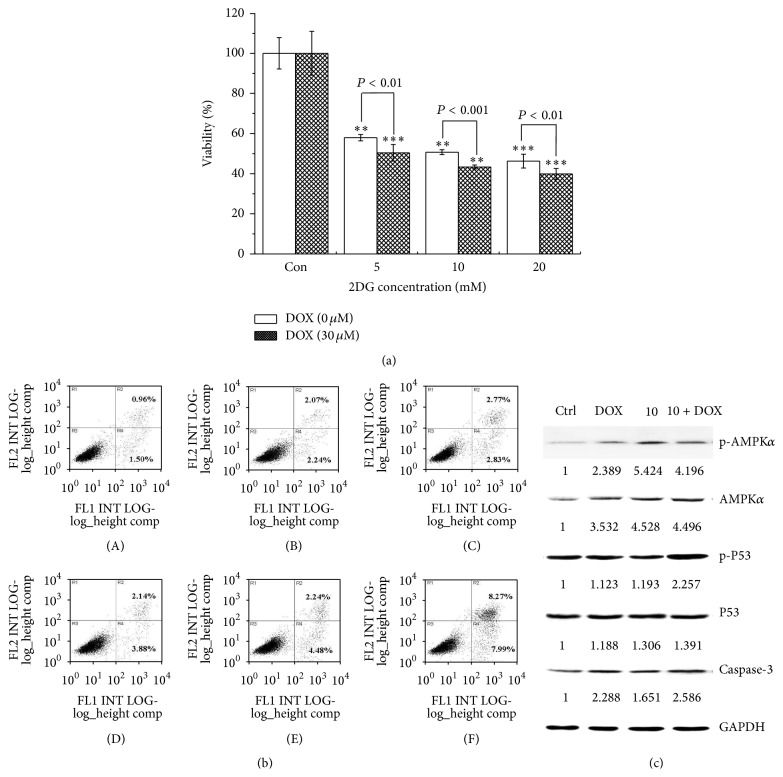
2DG sensitized MCF-7/MDR cells towards DOX. (a) The cytotoxicity of DOX was enhanced with the increase of 2DG concentration. ^*∗∗*^ *P* < 0.01; ^*∗∗∗*^ *P* < 0.001 versus control group. (b) Cell apoptosis was increased under the combination of 2DG and DOX. (A) The control group; (B) 10 mM 2DG group; (C) 20 mM 2DG group; (D) 30 *μ*M DOX; (E) the combination of 10 mM 2DG and 30 *μ*M DOX; (F) the combination of 20 mM 2DG and 30 *μ*M DOX. (c) The western blot assay was carried out after being exposed to either 2DG (10 mM) or DOX (30 *μ*M) or the combination of 2DG and DOX.

To verify the chemosensitized enhancement, the effect of 2DG on the cytotoxicity of DOX was investigated. We found that the 2DG was effective in enhancing the growth inhibition of 30 *μ*M DOX against MCF-7/MDR cells at even suboptimal concentration (5 mM) (Figure 3(a)). Meanwhile, the floating and adherent cells were harvested and stained with Annexin V-FITC for the analyses of apoptosis using flow cytometer. The apoptotic cell population comprised early apoptotic cells and those in the late stages. The results showed that the percentage of apoptotic cells induced by DOX was remarkably enhanced with the concentrations of 2DG. The percentage was almost increased by 3-fold from 6.02 ± 0.87% at single administration of 30 *μ*M DOX to 16.26 ± 0.39% at the combination of 20 mM 2DG and 30 *μ*M DOX (Figure 3(b)). To further explore the signal regulatory pathways, the protein levels of AMPK*α*, p53, and caspase-3 were analyzed by western blotting in total cell lysates and the result was shown in Figure 3(c). AMPK*α* had a very low expression in nontreated cells, but it would be accumulated in cells as cells were exposed to either 2DG or combination with DOX. In particular, 10 mM 2DG induced the significant activation of phospho-AMPK*α* no matter if cells received the administration of DOX or not. In addition, the level of phospho-p53 was increased by 2-fold and 1.89-fold in the combination group compared to those in response to DOX or 2DG single treatment group, respectively. Caspase-3 was a member of the cysteine-aspartic acid protease family and acted as an essential executive enzyme in the apoptosis signal pathway. As expected, its expression was increased after treatment with DOX. This increased activity was further augmented by addition of 2DG to reach a 1.13-fold expression, compared to DOX-treated cells. The finding was in concert with our previous apoptosis analysis and indicated that the combination of 2DG could further promote MCF-7/MDR cells apoptosis.

## 4. Discussion

Energy metabolism is the foundation of cell growth, proliferation, and differentiation. Cell proliferation relies on its demand of energy supply, particularly the status of intracellular ATP. The alteration of normal biochemical processes of glucose in cancer not only compensates the lack of growth nutrition factors, but also favors the tumor in the build-up of biomass more rapidly [9]. In human glioblastoma multiforme, the Warburg effect is crucial and correlated with worse overall survival of patients [10]. At present, targeting cancer cell metabolism provides a new promising strategy to preferentially kill the malignant cells.

2DG is a widely studied glucose analogue, which inhibits glucose metabolism by competitively inhibiting the uptake and utilization of glucose. 2DG is phosphorylated to generate 2DG-PO_4_ during the digestion. The latter is trapped in the cells and stops to be further metabolized to fulfill the inhibition of the glucose metabolism [11]. In our experiment, 2DG was used to inhibit the generation of ATP. The results presented that 2DG depleted the level of intercellular ATP and the generation of ATP was progressively lower with the increase in 2DG concentrations. Besides, AMPK*α* was activated and phosphorylated after cells were exposed to 2DG. AMPK*α* is a prominent sensor of cellular energy status, which acts as a marker of the level of intercellular ATP pool. 2DG decreased the level of ATP and increased the cellular AMP/ATP ratio, which inevitably led to the activation of AMPK*α* to promote catabolic processes and inhibit anabolic processes in response to ATP cellular demands [12]. Equally, we detected the depression of cell growth. It was postulated that 2DG affected the capacity of cell proliferation by depressing intercellular ATP.

Multidrug resistance is a phenomenon when cells acquire simultaneous resistance to certain chemotherapeutic agents, which frequently contribute to the overexpression of drug-efflux pump to reduce intracellular drug accumulation [13]. P-Glycoprotein (P-gp), multidrug resistance protein (MRP), and breast cancer resistance protein (BCRP), which are called the ATP-binding cassette (ABC) transporters, are a group of plasma membrane proteins that are associated with the development of drug resistance. Previous observations reported that the metabolic depletion was also accompanied by the potentiation of the depression of the drug-efflux and drug-sequestration systems [14]. Could the ATP depletion induced by 2DG chemosensitize and potentiate cytotoxic effect of DOX in MDR cells? The results showed that the drug-efflux systems were ATP-dependent, and a substantial mRNA decrease of P-gp and MRP1 was highly responsive to 2DG treatment. The less prominent activation of transmembrane transport system could retain the accumulation of DOX in cells, as seen in the Rh 123 accumulation assay. It is suggested that the inhibition on sensitization of DOX-resistant cell by P-gp and MRP1 was the result of 2DG-induced ATP depletion. Furthermore, the maintenance of pH gradients across organelle membranes was also essential for drug sequestration in MDR cells when the transportation was monitored by the activity of ATP-dependent pump [15]. It was observed that 2DG (10 *μ*M) significantly impaired the activity of Na^+^-K^+^-ATPase in MCF-7/MDR cells. Na^+^-K^+^-ATPase acts as a signal transducer/integrator to maintain resting potential, participating in transport and regulating cell energy expenditure. It provides the sodium gradient to import glucose, amino acids, and other nutrients into cells. For neurons, Na^+^-K^+^-ATPase is responsible for up to 2/3 of the cell's energy consumption [16]. When glucose metabolism is inhibited, the reduction in Na^+^-K^+^-ATPase activity is inevitable. In turn, the reduction in intracellular ATP levels might be potentiated by the repression of the levels of membrane transport system.

However, the depression of the activity of transmembrane transporters by 2DG was probably not the only mechanism involved in reversing the resistance of MCF-7/MDR cells to DOX. DOX is a feasible first-line chemotherapeutic agent, which induces DNA strand breakages by inhibiting the enzyme topoisomerase II. When DNA damage occurs, a large amount of ATP is required to form a dynamic repair complex at double-strand break sites [17]. In our previous studies, depletion of intracellular ATP retarded the repair of the potential DNA lethal damage [6]. It was suggested that 2DG depleting ATP pool and enhancing DNA damage might be a potential mechanism of the decreased DOX-induced cell viability by regulating the energy metabolism.

In conclusion, we propose the fact that 2DG reversed the resistance of MCF-7/MDR cells and enhanced DOX-induced apoptosis by interfering with the biochemical metabolism of glucose. The process was related to the depletion of intracellular ATP level, the inactivation of drug-efflux pump, and the depression of transmembrane transporters. Understanding energy metabolism in a combination of chemotherapeutic agents will provide the basis for rational suggestions of coadministration of energy metabolism inhibitor to maximize the killing of multidrug resistant cells.
